# Randomized trial comparing the effects of a 3D head-up system and microscope eyepiece-assisted simulated vitrectomy with intraocular illumination on the ocular surface of an operator

**DOI:** 10.1186/s12886-024-03516-4

**Published:** 2024-06-10

**Authors:** Xing Ge, Dandan Liu, Fangfang Fan, Tengyu Xu, Zhengpei Zhang, Haiyang Liu, Suyan Li

**Affiliations:** Department of Ophthalmology, The Affiliated Xuzhou Municipal Hospital of Xuzhou Medical University, Xuzhou First People’s Hospital, Xuzhou Eye Disease Prevention and Treatment Institute, Xuzhou, China

**Keywords:** 3D head-up system, Ocular surface, Tear meniscus height, Light intensity, Intraocular illumination, Vitrectomy

## Abstract

**Background:**

To compare the effects of a 3D head-up system and microscope eyepiece-assisted simulated vitrectomy intraocular illumination on the ocular surface of an operator.

**Methods:**

This was a prospective randomized controlled study. According to the application system, thirty ophthalmic operators (60 eyes) were randomly divided into 3D and eyepiece groups. Under different intensities of intraocular illumination, operators in both groups viewed the fundus model through a 3D display screen or microscopic eyepiece for 2 h. Objective examinations and a subjective symptom questionnaire were used immediately after the test to evaluate the ocular surface of the operators. Objective examinations included nonintrusion tear meniscus height (NIKTMH), nonintrusion break-up time (NIKBUT), and bulbar redness and strip meniscometry tube (SMTube) measurements. Statistical analyses were performed by using SPSS 26.0 software.

**Results:**

After the test, the NIKTMH, NIKBUT and SMTube measurements decreased; however, the degree of change varied among the groups of different systems. The differences between the 3D group and the eyepiece group in NIKTMH measurements, SMTube measurements, subjective symptom scores (eye dryness, difficulty focusing, and cervical pain), and light intensity reaching the ocular surface of the operators were statistically significant (*P* < 0.05). All of the objective and subjective tests showed that the 3D group had fewer effects on the NIKTMH and SMTube measurements, and the subjective comfort of the 3D group was greater.

**Conclusion:**

For both 3D screens and eyepieces, simulated vitrectomy with intraocular illumination for two hours can lead to discomfort and abnormalities in the operator’s ocular surface; however, these abnormalities are less severe in the 3D group.

**Trial registration:**

This trial was registered on December 22, 2022, at the Chinese Clinical Trials Registry with NO. ChiCTR2200066989.

**Supplementary Information:**

The online version contains supplementary material available at 10.1186/s12886-024-03516-4.

## Background

In previous studies, the advent of surgical microscope systems demonstrated the feasibility of intraocular surgery, which allows for operators to clearly observe the surgical field of the patient’s fundus and magnify it to perform internal eye surgery. With the rapid development of science and technology, digital video technology has been widely used in the field of ophthalmology, such as for three-dimensional (3D) head-up display systems, which provide operators with different surgical experience than microscopic eyepiece systems [1]. 3D head-up display systems capture the image signal in the surgical microscope system in real time through an High Data Registe (HDR) camera and send it to a 3D screen, where the operator views the stereoscopic images on the screen by wearing 3D glasses in a head-up position [2]. The 3D head-up system is an innovative technology for ophthalmic surgery. Unlike the traditional microscope eyepiece system, the 3D head-up system uses a high-definition digital display instead of an eyepiece. The operator changes the previous low surgical posture and wears 3D glasses to view the 3D display screen to obtain a visualized surgical field [3]. Many studies have confirmed that the 3D system has the following advantages. First, a clear operative field can be produced with a large magnification, wide field of view, and good depth of field, thus allowing for the operators to perform surgery with more apparent ocular structures. Additionally, the 3D head-up system allows for lower intraocular illumination, thus theoretically reducing medically induced retinal light damage. Moreover, the 3D head-up system allows for a head-up surgical posture, which is ergonomic and relaxes the muscles of the shoulder, neck, and lower back. Finally, the high-definition display allows for the operators and the assistants, students, and nursing staff involved in the procedure to observe the same surgical field, which is an advantage that is of utmost importance for medical teaching [4–10].

It has been shown that ophthalmologists inevitably experience a decrease in the blink rate when using slit lamps or surgical microscopes, which may lead to changes in tear secretion [11]. The 3D head-up system differs significantly from the microscope eyepiece system in terms of intraocular illumination, operative field presentation, working distance, and surgical posture. Our operators often perceive different ocular surface sensations when viewing a 3D screen and eyepiece during daily vitrectomy. Moreover, operators have reported of more pronounced subjective discomfort from ocular dryness when viewing the microscope eyepiece than when viewing the 3D display. Therefore, the two systems may affect the operators’ ocular surface in different ways. However, studies on the effects of 3D head-up system on the ocular surface of operators have yet to be reported. We designed and performed this study to investigate the differences in the effects of simulated vitrectomy and intraocular illumination on the ocular surface of operators under a 3D head-up system and a microscope eyepiece system. To assess the operators’ ocular surface and tear secretion, we used the oculus keratograph and the strip meniscometry tube (SMTube) for objective evaluations. Previously, only some studies have reported of the use of the SMTube measurement as a scientific indicator. The SMTube measurement is a new test paper for tear measurement that allows for the rapid testing of tear function in each eye within 5 s, and its accuracy and reproducibility of detection have been supported by the results of a previous study [12].

## Methods

This was a prospective randomized controlled study. This study followed the principles of the Declaration of Helsinki. The study was approved by the Medical Ethics Committee of Xuzhou First People’s Hospital (xyyll[2022]064) and registered with the Chinese Clinical Trials Registry (NO. ChiCTR2200066989). Each subject signed an informed consent form.

### Subjects

Volunteers were recruited to participate in this study. To ensure that the volunteers have similar physical health statuses and to exclude the influence of refractive status, ocular surface surgery, and diseases, among other factors, on the ocular surface parameters of volunteers, we established the following inclusion and exclusion criteria.

Inclusion criteria:

1. Young ophthalmic operators aged approximately 20–40 years.
2. Corrected visual acuity greater than or equal to 1.0 in both eyes.
3. Healthy ocular surface.

Exclusion criteria:

1. Both eyes underwent ocular surface surgery.
2. Differences in refractive power between the eyes were greater than 3D.
3. Wearing contact lenses.
4. Any ocular surface disease or systemic disease being observed with ocular surface complications.

The study recruited 30 ophthalmic operators at Xuzhou First People’s Hospital based on the sample size calculation results by using PASS software from December 2022 to February 2023. The staff responsible for recruitment recruited volunteers based on inclusion/exclusion criteria and numbered them in order of recruitment. The staff responsible for grouping used SPSS 26.0 software to generate random numbers for a completely randomized design grouping. Thirty volunteers were randomly divided into two groups, with 15 people in each group in a 1:1 allocation ratio. To ensure that grouping information was not leaked, the staff responsible for recruitment and the staff responsible for random grouping involved two different individuals. The staff responsible for assigning intervention measures requested that volunteers simulate vitrectomy for 2 h under a 3D head-up system and a microscopic eyepiece system. Fifteen (30 eyes) volunteers with a 3D head-up system were included in the 3D group, and 15 (30 eyes) volunteers with a microscope eyepiece system were included in the eyepiece group. Four males and 11 females in the 3D group had a mean age of 25.93 ± 2.60 years, and 3 males and 12 females in the eyepiece group had a mean age of 26.00 ± 2.17 years. There were no statistically significant differences observed between the two groups in terms of baseline age (*P* = 0.940), sex (*P* = 1.000), physician seniority (*P* = 1.000), refractive status (spherical mirror [*P* = 0.437], columnar mirror [*P* = 0.922]), ocular surface parameters (nonintrusion tear meniscus height [NIKTMH] [*P* = 0.717], nonintrusion break-up time [NIKBUT] [*P* = 0.552], bulbar redness [*P* = 0.666] or SMTube measurements [*P* = 0.335]) (Table 1).

**Table 1:** Comparison of population baseline data between 3D group and eyepiece group

	3D group	Eyepiece group	*P* value
Age (years)	25.93 ± 2.60	26.00 ± 2.17	0.940
Gender (male/female)	4/11	3/12	1.000
Physician seniority (junior/intermediate)	14/1	13/2	1.000
Spherical mirror (degree)	-0.50(-1.13,0.00)	-0.75(-1.50,0.00)	0.437
Columnar mirror (degree)	0.00(-0.50,0.00)	0.00(-0.50,0.00)	0.922
NIKTMH(mm)	0.20(0.19,0.20)	0.20(0.17,0.23)	0.717
NIKBUT(s)	11.410 ± 3.64	10.82 ± 4.00	0.552
Bulbar Redness(points)	0.80(0.60,0.80)	0.85(0.50,0.90)	0.666
SMTube measurement(mm)	8.00(5.00,9.00)	7.00(6.75,8.00)	0.335

NIKTMH: non-intrusion tear meniscus height; NIKBUT: non-intrusion break-up time; SMTube: Strip Meniscometry Tube.

### Environment and equipment

**Fig. 1:**
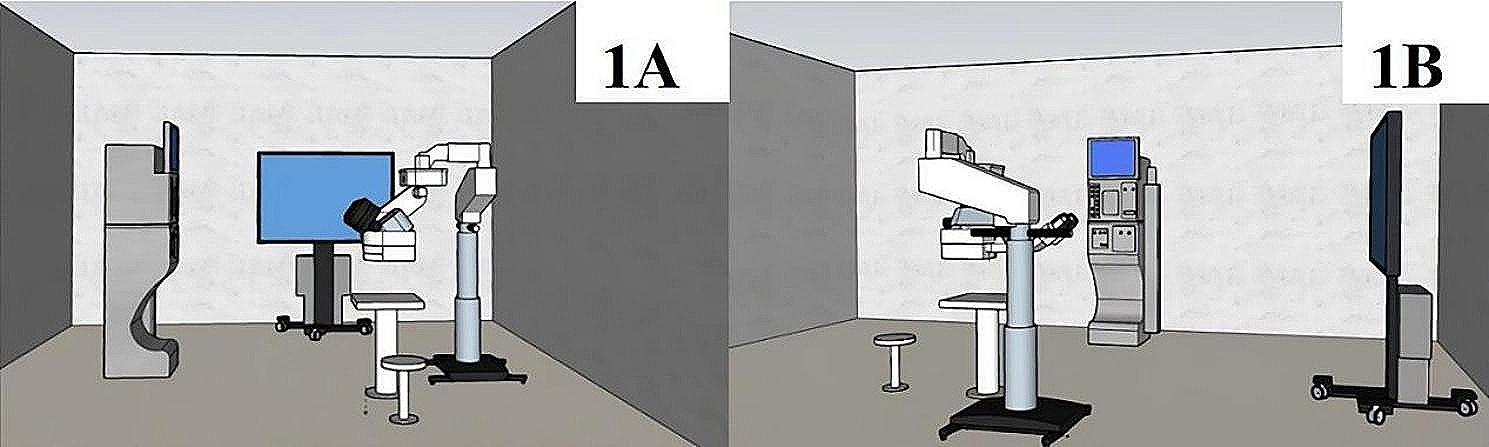
Schematic diagram of the test environment and equipment placement. Figure 1A is a front view. Figure 1B is a side view

**Fig. 2:**
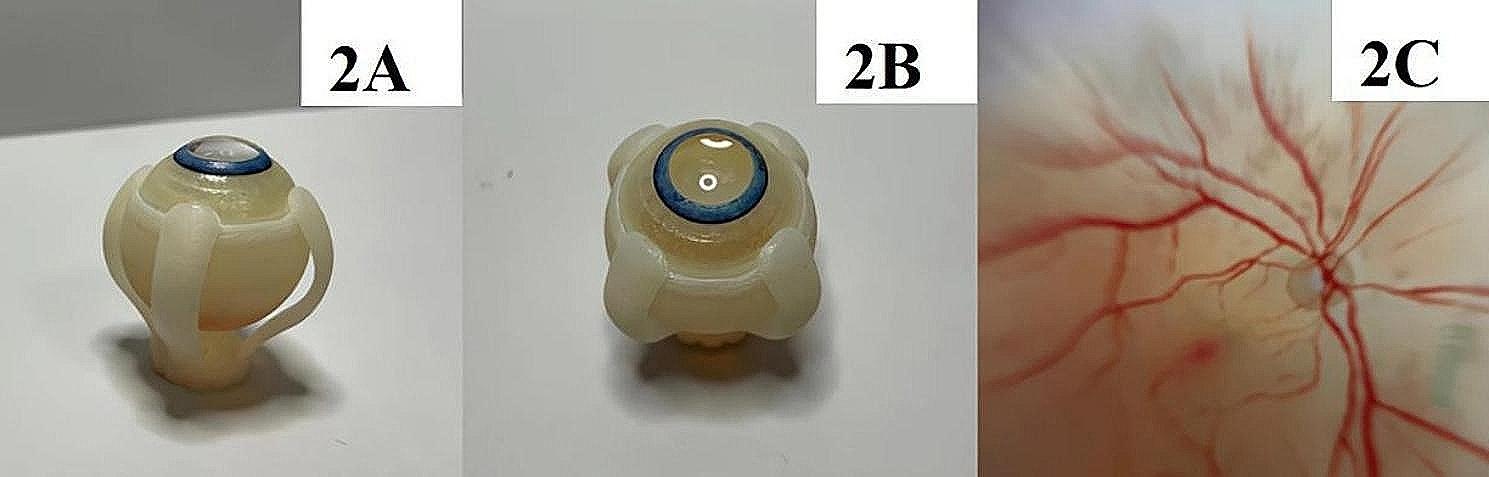
Schematic diagram of the eye model. Figure 2A shows a side view of the model appearance. Figure 2B shows a top view of the model appearance. Figure 2C shows a detailed view of the eye fundus inside of the model

The trial environment and equipment placement are shown in Fig. 1. The environmental scenario, equipment installation, data setting and observation duration (2 h) of the test were set according to our previous clinical standard of conventional vitrectomy. The test was performed in a dark room to eliminate interference from light sources other than the illumination system. Through the control system, we controlled the temperature inside of the house at 25 °C and the humidity at 50%. The utilized test equipment was the NGENUITY3D visualization surgical system (Alcon, USA), a noncontact wide-angle microsurgical scope (Zeiss, Germany) and a vitrectomy machine (Alcon, USA). The microscope eyepiece system, the vitrectomy machine, and the operator’s operating table were placed at the same level. The 3D display screen was placed 1.8 m in front of the operator’s direct vision, with the operator’s direct vision at the bottom 1/3 of the screen. The simulated vitrectomy was performed on an eye model (Alcon, USA) (Fig. 2) for 2 h by each operator. The size of the eye model was based on a natural human eye, with a transparent structure at the top and a structure simulating the human fundus at the bottom of the inner surface. The intraocular illumination system uses high-brightness LED light sources, which can enable surgeons to clearly observe the structure inside of the eye. The light source also has good colour temperature and colour reproduction. Moreover, the light source of the intraocular illumination system can adjust the brightness, and doctors can adjust the brightness as needed to achieve the best observation effect. The intraocular illumination system (optical fibres and chandeliers) was attached to the vitrectomy machine at one end and inserted inside of the eye model through the trocar at the other end to illuminate the fundus structures. The parameter sets of simulated vitrectomy intraocular illumination were based on the data of the previous clinical application of the two systems for vitrectomy. The optical fibres were set at 15%, and the chandeliers were set at 15% in the 3D group. The percentage of optical fibres was set at 46%, and the percentage of chandeliers was set at 32% in the eyepiece group. The actual light intensity was measured with a photometer (TES1330A, China).

### Test procedure

**Fig. 3:**
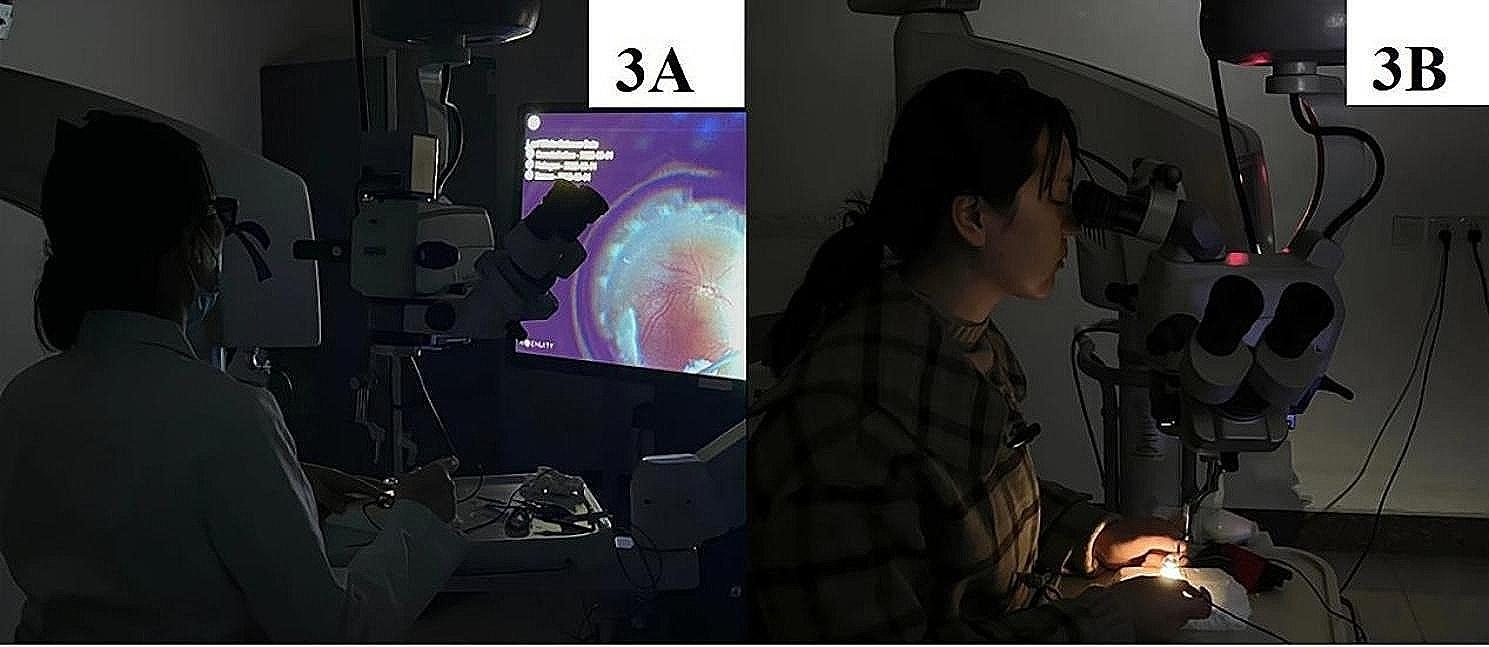
Schematic diagram of the test performed by the operators. Figure 3A shows that an operator in the 3D group was performing an operation. Figure 3B shows that an operator in the eyepiece group was performing an operation

In the test, operators were asked to apply the specified system and intraocular illumination parameters for viewing the simulated fundus’s visual field. The operators were given a regular and healthy routine before the beginning of the test and did not use any medication that affects the eye surface, including any eye drops, alcohol, or caffeine, among other medications; additionally, baseline data on ocular surface parameters were collected by the researcher responsible for measurements and examination. Moreover, all of the light sources were turned off after the operators entered the test site to maintain a dark environment, and the operators applied a 3D head-up system or a microscope eyepiece system according to grouping. The optical fibres and chandeliers were inserted into the eye model, and the researcher was responsible for implementing interventions that adjusted the parameters to the specified intensity of the intraocular illumination system. In addition, operators in the 3D group wearing 3D glasses viewed the 3D display screen (Fig. 3), and operators in the eyepiece group viewed the eyepiece directly (Fig. 3); furthermore, operators in each group used an inverted mirror to obtain the fundus image in the eye model by viewing the 3D display screen or eyepiece. The intensity of light reaching the ocular surface of the operators was measured by the researcher responsible for measurements and examinations by using a photometer. After two hours of viewing (from 8 am to 10 am), operators were immediately asked to perform examinations of ocular surface parameters and to complete a subjective symptom questionnaire. All of the examinations were performed three times by the researcher responsible for measurements and examinations to calculate an average value. The personnel responsible for the measurements and statistical analysis were unaware of the grouping and intervention conditions.

### Objective examination of ocular surface parameters

Oculus keratography (OCULUS, Germany) was used to evaluate the nonintrusion tear meniscus height (NIKTMH), nonintrusion break-up time (NIKBUT) and bulbar redness of the operators. The tear river was observed through high-definition images. We determined the height of the tear river in the lower eyelid and recorded the displayed value. A normal NIKTMH value was calculated to be ≥ 0.20 mm. The Oculus Keratograph Integrated Analyser measures the NIKBUT through a noninvasive, automated, objective quantification technique that avoids manual timing and subjective errors. The standard value of the NIKBUT is 10–45 s. Bulbar redness was measured by this instrument to quantify the degree of conjunctival and ciliary blood congestion. The general standard value of the Bulbar redness score was calculated to be < 1.0 [13]. The tear secretion of the operators was measured via the SMTube (Echo Electricity Co., Ltd., Japan). When the test paper touches the tear river in the lower lid of an eye, the paper adsorbs the tear via the capillary phenomenon, the tear moves up the groove of the filter paper, and the length of the blue indicator on the filter paper after 5 s of contact with the tear river indicates the result of the measurement [12].

### Subjective symptoms questionnaire

The subjective symptoms questionnaire was designed based on a literature review of ocular surface disease questionnaires and a study by Seguí Mdel et al. [14]. The questionnaire included 14 subjective symptoms related to dry eye symptoms (burning sensation, foreign body sensation, excessive blinking, tearing, dryness, eye pain, and photophobia), visual disturbances (blurred vision, diplopia, and difficulty with focusing) and neurological symptoms (headache, dizziness, nausea, and cervical pain). The operators were assessed for subjective symptoms prior to the beginning of the test, and no operators reported of any related symptoms. At the end of the test, operators were rated on a 6-point scale according to the severity of symptoms (scale: 0 = none, 1 = minor symptoms, 2 = symptoms but not significant, 3 = significant symptoms, 4 = severe symptoms, and 5 = severe to overwhelming).

### Statistical analysis

Statistical analyses were performed by using SPSS 26.0 software (IBM, USA). The Shapiro‒Wilk test was used to determine the normality of the measurement data. Normally distributed variables are expressed as the mean ± standard deviation. Differences in the measured parameters before and after the operation were evaluated by using paired t tests, and the intergroup differences were compared by using independent sample t tests. Nonnormally distributed data are expressed as medians and interquartile ranges (M [Q1, Q3]), and the Wilcoxon signed-rank test was used for intragroup difference evaluation, whereas the Mann‒Whitney U test was used for intergroup difference evaluation. Count data are expressed as frequencies and percentages, and differences between two groups were compared by using the chi-square χ2 test. Fisher’s exact test was used when the theoretical frequency was < 0.05. When the test level was α = 0.05, *P* < 0.05 was considered to indicate a statistically significant difference.

## Results

### Comparison of ocular surface parameters between the 3D group and eyepiece group after the test

After the test, the NIKTMH (*P* = 0.001) and SMTube (*P* = 0.004) measurements in the 3D group were significantly better than those in the eyepiece group, and the differences were statistically significant. No significant differences were found in NIKBUT (*P* = 0.051) or bulbar redness (*P* = 0.730) between the two groups (Table 2).

**Table 2:** Comparison of ocular surface parameters after the test between 3D group and eyepiece group

Ocular Surface Parameters	3D group (*n* = 30)	Eyepiece group(*n* = 30)	*P* value
NIKTMH(mm)	0.18(0.16,0.18)	0.14(0.13,0.14)	0.001
NIKBUT(s)	8.91 ± 4.21	6.94 ± 3.40	0.051
Bulbar Redness(points)	0.80(0.70,0.80)	0.90(0.60,1.00)	0.730
SMTube measurement(mm)	6.43 ± 2.27	4.83 ± 1.84	0.004

NIKTMH: non-intrusion tear meniscus height; NIKBUT: non-intrusion break-up time; SMTube: Strip Meniscometry Tube.

### Comparison of ocular surface parameters before and after the test in the 3D group

The ocular surface parameters were compared before and after surgery for operators in the 3D group. The differences in the NIKTMH (*P* < 0.001), NIKBUT (*P* < 0.001), and SMTube measurements (*P* < 0.001) before and after the test were statistically significant, and the values before the test were significantly greater than those after the test. The difference in the bulbar redness scores (*P* = 0.192) before and after the test was not statistically significant (Table 3).

**Table 3:** Comparison of ocular surface parameters before and after test in 3D group

Ocular Surface Parameters	Befor test (*n* = 30)	After test (*n* = 30)	*P* value
NIKTMH(mm)	0.20(0.19,0.20)	0.18(0.16,0.18)	< 0.001
NIKBUT(s)	11.41 ± 3.64	8.91 ± 4.21	< 0.001
Bulbar Redness(points)	0.80(0.60,0.80)	0.80(0.70,0.80)	0.192
SMTube measurement(mm)	8.30 ± 2.20	6.43 ± 2.27	< 0.001

NIKTMH: non-intrusion tear meniscus height; NIKBUT: non-intrusion break-up time; SMTube: Strip Meniscometry Tube.

### Comparison of ocular surface parameters before and after the test in the eyepiece group

The ocular surface parameters were compared before and after the test for operators in the eyepiece group. The differences in the NIKTMH (*P* < 0.001), NIKBUT (*P* < 0.001), and SMTube measurements (*P* < 0.001) before and after the test were statistically significant, and the values before the test were significantly greater than those after the test. The difference in bulbar redness before and after the test was not statistically significant (*P* = 0.121) (Table 4).

**Table 4:** Comparison of ocular surface parameters before and after test in eyepiece group

Ocular Surface Parameters	Befor test (*n* = 30)	After test (*n* = 30)	*P* value
NIKTMH(mm)	0.20(0.17,0.23)	0.14(0.13,0.14)	< 0.001
NIKBUT(s)	10.82 ± 4.00	6.94 ± 3.40	< 0.001
Bulbar Redness(points)	0.85(0.50,0.90)	0.90(0.60,1.00)	0.121
SMTube measurement(mm)	7.00(6.75,8.00)	4.50(3.75, 6.00)	< 0.001

NIKTMH: non-intrusion tear meniscus height; NIKBUT: non-intrusion break-up time; SMTube: Strip Meniscometry Tube.

### Comparison of subjective symptom points between the 3D group and eyepiece group after the test

Regarding subjective symptoms, the operators in the 3D group had significantly lower eye dryness (*P* < 0.001), difficulty with focusing (*P* = 0.029), cervical pain (*P* < 0.001), and total score (*P* < 0.001) measurements than did those in the eyepiece group. No statistically significant differences were found between the two groups in terms of eye-burning sensation (*P* = 0.775), foreign body sensation (*P* = 0.217), excessive blinking (*P* = 0.174), lacrimation (*P* = 0.061), eye pain (*P* = 0.305), photophobia (*P* = 0.217), blurred vision (*P* = 0.202), diplopia (*P* = 0.775), headache (*P* = 0.539), dizziness (*P* = 0.539) or nausea (*P* = 0.775) (Table 5).

**Table 5:** Comparison of subjective symptom questionnaires between 3D group and eyepiece group after test

Subjective symptom (points)	3D group (*n* = 15)	Eyepiece group (*n* = 15)	*P* value
Burning sensation	1.00(1.00,1.00)	1.00(1.00,1.00)	0.775
Foreign body sensation	1.00(1.00,1.00)	1.00(1.00,2.00)	0.217
Excessive blinking	1.00(1.00,1.00)	1.00(1.00,3.00)	0.174
Lacrimation	1.00(1.00,1.00)	1.00(1.00,2.00)	0.061
Dryness	1.00(1.00,1.00)	3.00(2.00,3.00)	< 0.001
Eye pain	1.00(1.00,1.00)	1.00(1.00,2.00)	0.305
Photophobia	1.00(1.00,1.00)	1.00(1.00,2.00)	0.217
Blurred vision	1.00(1.00,1.00)	1.00(1.00,2.00)	0.202
Diplopia	0.00(0.00,0.00)	0.00(0.00,0.00)	0.775
Difficulty focusing	1.00(1.00,1.00)	1.00(1.00,2.00)	0.029
Headache	0.00(0.00,0.00)	0.00(0.00,0.00)	0.539
Dizziness	0.00(0.00,0.00)	0.00(0.00,1.00)	0.539
Nausea (points)	0.00(0.00,0.00)	0.00(0.00,0.00)	0.775
Cervical pain	0.0(0.00,0.00)	1.00(0.00,2.00)	< 0.001
Total score	11.00(9.00,11.00)	15.0(13.00,18,00)	< 0.001

### Comparison of the light intensity reaching the ocular surface between the 3D group and eyepiece group during simulated vitrectomy intraocular illumination

The light intensity reaching the operators’ ocular surface in the 3D group was 3.21 ± 0.61 lx, and the light intensity reaching the operators’ ocular surface in the eyepiece group was 17.87 ± 2.72 lx. The light intensity reaching the operators’ eye surface in the 3D group was significantly lower than that in the eyepiece group, and the difference between the two groups was statistically significant (*P* < 0.001).

## Discussion

Our study showed that both the application of the 3D head-up system and the application of the microscope eyepiece system affected the operators’ ocular surface during simulated vitrectomy intraocular illumination. After 2 h of viewing the 3D display screen or eyepiece, the reductions in the NIKTMH, NIKBUT, and SMTube measurements indicated that tear secretion and tear dynamics were impaired regardless of the system that was applied to simulate vitrectomy intraocular illumination. Therefore, we conclude that operators also experience alterations in ocular surface stability when performing actual vitrectomy. However, in daily clinical practice, the cumulative time to perform vitrectomy is much longer than 2 h, which may increase the risk of abnormal ocular surface tissue. Previous studies have shown that the professional activities that ophthalmologists engage in for long time periods, including the application of computers, microscopes and slit lamps, can decrease blink frequency [11]. Yan et al. showed that increasing amounts of high-intensity work performed by ophthalmologists lead to considerable alterations in tear dynamics, which can cause dry eye [15]. Therefore, occupational ocular surface damage in the field of ophthalmology is a concern.

In our test, although the use of both the 3D head-up system and the microscope eyepiece system affected the operator’s ocular surface, the NIKTMH and SMTube measurements were significantly better in the 3D group than in the eyepiece group after the test. Compared with the eyepiece group, the 3D head-up system had less effect on the operator’s tear secretion, thus suggesting that the application of the 3D head-up system for vitrectomy may have reduced the abnormality of the ocular surface stability of the operators. Dzhodzhua et al. suggested that temperature, humidity, and illumination in the working environment of ophthalmologists can have visual effects [11]. Zakerian et al. also noted that different lighting intensities could affect vision in different ways [16]. In our study, the 3D head-up system with its high-definition magnified display effectively reduced the light intensity of the intraocular illumination system, and operators could further reduce the light intensity reaching the ocular surface by wearing 3D glasses. The light intensity reaching the operators’ ocular surface measured via the photometer in the 3D group was significantly lower than that in the eyepiece group. To a certain extent, the 3D head-up system reduces the risk of light damage to the operator’s ocular surface. In contrast, operators in the eyepiece group had a significantly greater light intensity reaching their ocular surface through the surgical field, as obtained by direct observation of the eyepiece, than did those in the 3D group. The different light intensities of the two systems may be one of the reasons for the disparity in the altered tear secretion of the subjects. In addition, the 3D head-up system has a high-definition magnified display screen, and the operators can still see the surgical field at a distance of 1.8 m from the 3D display screen. Compared to the relatively limited surgical field of view and the shorter working distance of the eyepiece system, the operators are more relaxed when applying the 3D head-up system for vitrectomy. The relatively long working distance and the clarity of the surgical field of view also correlated with differences in ocular surface tear secretion between the two groups. Previous studies agree that excessive eye use due to close working distance has more severe consequences, thus inevitably causing a decrease in blink frequency and changes in tear secretion [11]. A study by Viktoriya et al. also indicated that a close working distance reduces accommodation, convergence and tear secretion, with implications for ocular surface comfort in subjects [11]. Although the causes of ocular surface discomfort and injury in operators cannot be identified, we believe that light intensity is the main cause, whereas working distance and field size are the secondary causes.

Subjective questionnaires are the most commonly used assessment method for research. However, they are often evaluated based on the subjective feelings and symptoms of the participant, which often vary from person to person. Therefore, this assessment method is only used as a reference and requires more precision. In this study, operators in the eyepiece group had higher overall scores on the subjective questionnaire than did those in the 3D group, and operators in this group experienced more subjective discomfort when viewing the eyepiece. These subjective discomfort characteristics mainly included dryness, difficulty with focusing and cervical pain. Although subjective questionnaires are often considered a method of assessment that lacks objectivity, it has been suggested that the mental fatigue caused by subjective sensory discomfort can correspondingly affect visual fatigue [11]. Therefore, operators’ subjective perceptions are important and should not be neglected, and relaxed and comfortable subjective perceptions and mental states positively impact visual health. Subjects who use microscope eyepiece systems are more likely to experience subjective discomfort. According to a questionnaire survey by Flavin et al., 56% of pathology laboratory personnel suffer from microscopy-related visual problems, including eye fatigue, discomfort, headache, dry eyes, dizziness and nausea [17]. A similar questionnaire survey was conducted by Jain et al., in which 94% of subjects reported of symptoms related to eye discomfort, including eye discomfort, headache and dry eyes, and the vast majority of subjects expressed ocular discomfort while using the microscope [18]. Moreover, operators working with a microscope under high workload for an extended period of time experienced ocular and mental discomfort. Based on our findings, the use of a 3D head-up system may result in more comfortable ocular sensations than the use of a microscope eyepiece system, which (to some extent) is beneficial for the protection of visual health.

There were still some limitations of this study. First, the sample size was small, and the test length was short because operators work more than 2 h daily. Second, to ensure a similar physical health status, we chose operators aged 20 to 35 years, which cannot completely represent operators of different age groups. Additionally, to control for variables, we chose to have both groups of operators observe the same fundus model for the simulated operation; however, this was not truly representative of the actual vitrectomy procedure. Finally, although the test design has limitations, the factors affecting the operators’ ocular surface conditions are multifaceted and cannot be avoided. Although this study had some limitations, it still has significant advantages and influence. Previously, there were few reports on the impact of a 3D head-up system on the ocular surface of operators. Our research can provide directions and references for future research.

## Conclusion

The study results indicate that the operator’s tear secretion was indeed impaired when performing the simulated operation. From this result, we can infer that vitrectomy may impact the ocular surface stability of operators. However, compared with the microscope eyepiece system, the 3D head-up system, with its advantages of low intraocular illumination, long distance, and high-definition field of view, has less impact on the operators’ tear secretion. The 3D head-up system may provide some protection to the ocular surface and increase personal comfort for operators.

### Electronic supplementary material

Below is the link to the electronic supplementary material.

Supplementary Material 1

Supplementary Material 2

## Data Availability

The datasets generated and analyzed during the current study, as well as the trial protocol are available from the corresponding author on reasonable request.
