# Supplementary material for: Randomized trial comparing the effects of a 3D head-up system and microscope eyepiece-assisted simulated vitrectomy with intraocular illumination on the ocular surface of an operator
Source: BMC Ophthalmol. 2024 Jun 10;24:241. doi: 10.1186/s12886-024-03516-4 (PMC11163792; doi:10.1186/s12886-024-03516-4)
Supplement: Supplementary file 2 — Supplementary Material 2 [file 12886_2024_3516_MOESM2_ESM.docx]

**Randomized trial comparing the effects of a 3D head-up system and microscope eyepiece-assisted simulated vitrectomy with intraocular illumination on the ocular surface of an operator**

**Clinical trial protocol**

**Protocol abstract**

| Name | Randomized trial comparing the effects of a 3D head-up system and microscope eyepiece-assisted simulated vitrectomy with intraocular illumination on the ocular surface of an operator |
| --- | --- |
| Objectives | To compare the differences in the effects of 3D head-up system and microscope eyepiece assisted simulated vitrectomy intraocular illumination on the ocular surface of the operator |
| Design | A prospective randomized controlled study |
| Volunteers | 30 volunteers |
| Selection criteria | 1. Inclusion criteria: 2. Young ophthalmic operators aged approximately 20-40 years 3. Corrected visual acuity greater than or equal to 1.0 in both eyes 4. Healthy ocular surface |
|  | Exclusion criteria:   1. Both eyes underwent ocular surface surgery 2. Differences in refractive power between the eyes were greater than 3D 3. Wearing contact lenses 4. Any ocular surface disease or systemic disease being observed with ocular surface complications |
| Methodology | This is a prospective randomized controlled study. According to the application system, thirty ophthalmic operators (60 eyes) were randomly divided into 3D group and eyepiece group. Under different light intensity of the intraocular illumination, operators in both groups viewed the fundus model through the 3D display screen or microscopic eyepiece for 2 hours, respectively. Objective examinations and a subjective symptom questionnaire were used immediately after test to evaluate the ocular surface of the operators. Objective examinations included non-intrusion tear meniscus height (NIKTMH), non-intrusion break-up time (NIKBUT), bulbar redness and Strip Meniscometry Tube (SMTube) measurements. Statistical analyses were performed using SPSS 26.0 software. |
| Statistics | Statistical analyses were performed by using SPSS 26.0 software (IBM, USA). The Shapiro‒Wilk test was used to determine the normality of the measurement data. Normally distributed variables are expressed as the mean ± standard deviation. Differences in the measured parameters before and after the operation were evaluated by using paired t tests, and the intergroup differences were compared by using independent sample t tests. Nonnormally distributed data are expressed as medians and interquartile ranges (M [Q1, Q3]), and the Wilcoxon signed-rank test was used for intragroup difference evaluation, whereas the Mann‒Whitney U test was used for intergroup difference evaluation. Count data are expressed as frequencies and percentages, and differences between two groups were compared by using the chi-square χ2 test. Fisher's exact test was used when the theoretical frequency was < 0.05. When the test level was α= 0.05, P < 0.05 was considered to indicate a statistically significant difference. |
| Organization | Department of Ophthalmology, The Affiliated Xuzhou Municipal Hospital of Xuzhou Medical University, Xuzhou First People's Hospital, Xuzhou Eye Disease Prevention and Treatment Institute, Xuzhou, China |

**Background**

In previous studies, the advent of surgical microscope systems demonstrated the feasibility of intraocular surgery, which allows for operators to clearly observe the surgical field of the patient's fundus and magnify it to perform internal eye surgery. With the rapid development of science and technology, digital video technology has been widely used in the field of ophthalmology, such as for three-dimensional (3D) head-up display systems, which provide operators with different surgical experience than microscopic eyepiece systems [1]. 3D head-up display systems capture the image signal in the surgical microscope system in real time through an High Data Registe (HDR) camera and send it to a 3D screen, where the operator views the stereoscopic images on the screen by wearing 3D glasses in a head-up position. The 3D head-up system is an innovative technology for ophthalmic surgery. Unlike the traditional microscope eyepiece system, the 3D head-up system uses a high-definition digital display instead of an eyepiece. The operator changes the previous low surgical posture and wears 3D glasses to view the 3D display screen to obtain a visualized surgical field. Many studies have confirmed that the 3D system has the following advantages. First, a clear operative field can be produced with a large magnification, wide field of view, and good depth of field, thus allowing for the operators to perform surgery with more apparent ocular structures. Additionally, the 3D head-up system allows for lower intraocular illumination, thus theoretically reducing medically induced retinal light damage. Moreover, the 3D head-up system allows for a head-up surgical posture, which is ergonomic and relaxes the muscles of the shoulder, neck, and lower back. Finally, the high-definition display allows for the operators and the assistants, students, and nursing staff involved in the procedure to observe the same surgical field, which is an advantage that is of utmost importance for medical teaching.

It has been shown that ophthalmologists inevitably experience a decrease in the blink rate when using slit lamps or surgical microscopes, which may lead to changes in tear secretion. The 3D head-up system differs significantly from the microscope eyepiece system in terms of intraocular illumination, operative field presentation, working distance, and surgical posture. Our operators often perceive different ocular surface sensations when viewing a 3D screen and eyepiece during daily vitrectomy. Moreover, operators have reported of more pronounced subjective discomfort from ocular dryness when viewing the microscope eyepiece than when viewing the 3D display. Therefore, the two systems may affect the operators' ocular surface in different ways. However, studies on the effects of 3D head-up systems on the ocular surface of operators have yet to be reported. We designed and performed this study to investigate the differences in the effects of simulated vitrectomy and intraocular illumination on the ocular surface of operators under a 3D head-up system and a microscope eyepiece system. To assess the operators' ocular surface and tear secretion, we used the oculus keratograph and the strip meniscometry tube (SMTube) for objective evaluations. Previously, only some studies have reported of the use of the SMTube measurement as a scientific indicator. The SMTube measurement is a new test paper for tear measurement that allows for the rapid testing of tear function in each eye within 5 seconds, and its accuracy and reproducibility of detection have been supported by the results of a previous study.

**Objectives**

To compare the differences in the effects of 3D head-up system and microscope eyepiece assisted simulated vitrectomy intraocular illumination on the ocular surface of the operator.

**Design**

This is a prospective randomized controlled study.

**Methodology**

**Subjects**

Volunteers were recruited to participate in this study.

Inclusion criteria:

1. Young ophthalmic operators aged approximately 20-40 years
2. Corrected visual acuity greater than or equal to 1.0 in both eyes
3. Healthy ocular surface

Exclusion criteria:

1. Both eyes underwent ocular surface surgery
2. Differences in refractive power between the eyes were greater than 3D
3. Wearing contact lenses
4. Any ocular surface disease or systemic disease being observed with ocular surface complications

The study recruited 30 ophthalmic operators at Xuzhou First People's Hospital based on the sample size calculation results by using PASS software from December 2022 to February 2023. The staff responsible for recruitment recruited volunteers based on inclusion/exclusion criteria and numbered them in order of recruitment. The staff responsible for grouping used SPSS 26.0 software to generate random numbers for a completely randomized design grouping. Thirty volunteers were randomly divided into two groups, with 15 people in each group in a 1:1 allocation ratio. To ensure that grouping information was not leaked, the staff responsible for recruitment and the staff responsible for random grouping involved two different individuals. The staff responsible for assigning intervention measures requested that volunteers simulate vitrectomy for 2 hours under a 3D head-up system and a microscopic eyepiece system. Fifteen (30 eyes) volunteers with a 3D head-up system were included in the 3D group, and 15 (30 eyes) volunteers with a microscope eyepiece system were included in the eyepiece group. Four males and 11 females in the 3D group had a mean age of 25.93 ± 2.60 years, and 3 males and 12 females in the eyepiece group had a mean age of 26.00 ± 2.17 years. There were no statistically significant differences observed between the two groups in terms of baseline age (P = 0.940), sex (P =1.000), physician seniority (P = 1.000), refractive status (spherical mirror [P = 0.437], columnar mirror [P = 0.922]), ocular surface parameters (nonintrusion tear meniscus height [NIKTMH] [P = 0.717], nonintrusion break-up time [NIKBUT] [P = 0.552], bulbar redness [P = 0.666] or SMTube measurements [P = 0.335]).

**Environment and equipment**

The trial environment and equipment placement are shown in Figure 1. The environmental scenario, equipment installation, data setting and observation duration (2 hours) of the test were set according to our previous clinical standard of conventional vitrectomy. The test was performed in a dark room to eliminate interference from light sources other than the illumination system. Through the control system, we controlled the temperature inside of the house at 25 °C and the humidity at 50%. The utilized test equipment was the NGENUITY3D visualization surgical system (Alcon, USA), a noncontact wide-angle microsurgical scope (Zeiss, Germany) and a vitrectomy machine (Alcon, USA). The microscope eyepiece system, the vitrectomy machine, and the operator's operating table were placed at the same level. The 3D display screen was placed 1.8 m in front of the operator's direct vision, with the operator's direct vision at the bottom 1/3 of the screen. The simulated vitrectomy was performed on an eye model (Alcon, USA) for 2 hours by each operator. The size of the eye model was based on a natural human eye, with a transparent structure at the top and a structure simulating the human fundus at the bottom of the inner surface. The intraocular illumination system uses high-brightness LED light sources, which can enable surgeons to clearly observe the structure inside of the eye. The light source also has good colour temperature and colour reproduction. Moreover, the light source of the intraocular illumination system can adjust the brightness, and doctors can adjust the brightness as needed to achieve the best observation effect. The intraocular illumination system (optical fibres and chandeliers) was attached to the vitrectomy machine at one end and inserted inside of the eye model through the trocar at the other end to illuminate the fundus structures. The parameter sets of simulated vitrectomy intraocular illumination were based on the data of the previous clinical application of the two systems for vitrectomy. The optical fibres were set at 15%, and the chandeliers were set at 15% in the 3D group. The percentage of optical fibres was set at 46%, and the percentage of chandeliers was set at 32% in the eyepiece group. The actual light intensity was measured with a photometer (TES1330A, China).

**Test procedure**

In the test, operators were asked to apply the specified system and intraocular illumination parameters for viewing the simulated fundus's visual field. The operators were given a regular and healthy routine before the beginning of the test and did not use any medication that affects the eye surface, including any eye drops, alcohol, or caffeine, among other medications; additionally, baseline data on ocular surface parameters were collected by the researcher responsible for measurements and examination. Moreover, all of the light sources were turned off after the operators entered the test site to maintain a dark environment, and the operators applied a 3D head-up system or a microscope eyepiece system according to grouping. The optical fibres and chandeliers were inserted into the eye model, and the researcher was responsible for implementing interventions that adjusted the parameters to the specified intensity of the intraocular illumination system. In addition, operators in the 3D group wearing 3D glasses viewed the 3D display screen, and operators in the eyepiece group viewed the eyepiece directly; furthermore, operators in each group used an inverted mirror to obtain the fundus image in the eye model by viewing the 3D display screen or eyepiece. The intensity of light reaching the ocular surface of the operators was measured by the researcher responsible for measurements and examinations by using a photometer. After two hours of viewing (from 8 am to 10 am), operators were immediately asked to perform examinations of ocular surface parameters and to complete a subjective symptom questionnaire. All of the examinations were performed three times by the researcher responsible for measurements and examinations to calculate an average value. The personnel responsible for the measurements and statistical analysis were unaware of the grouping and intervention conditions.

**Objective examination of ocular surface parameters**

Oculus keratography (OCULUS, Germany) was used to evaluate the nonintrusion tear meniscus height (NIKTMH), nonintrusion break-up time (NIKBUT) and bulbar redness of the operators. The tear river was observed through high-definition images. We determined the height of the tear river in the lower eyelid and recorded the displayed value. A normal NIKTMH value was calculated to be ≥ 0.20 mm. The Oculus Keratograph Integrated Analyser measures the NIKBUT through a noninvasive, automated, objective quantification technique that avoids manual timing and subjective errors. The standard value of the NIKBUT is 10-45 seconds. Bulbar redness was measured by this instrument to quantify the degree of conjunctival and ciliary blood congestion. The general standard value of the Bulbar redness score was calculated to be < 1.0. The tear secretion of the operators was measured via the SMTube (Echo Electricity Co., Ltd., Japan). When the test paper touches the tear river in the lower lid of an eye, the paper adsorbs the tear via the capillary phenomenon, the tear moves up the groove of the filter paper, and the length of the blue indicator on the filter paper after 5 seconds of contact with the tear river indicates the result of the measurement.

**Subjective symptoms questionnaire**

The subjective symptoms questionnaire was designed based on a literature review of ocular surface disease questionnaires and a study by Seguí Mdel et al. [14]. The questionnaire included 14 subjective symptoms related to dry eye symptoms (burning sensation, foreign body sensation, excessive blinking, tearing, dryness, eye pain, and photophobia), visual disturbances (blurred vision, diplopia, and difficulty with focusing) and neurological symptoms (headache, dizziness, nausea, and cervical pain). The operators were assessed for subjective symptoms prior to the beginning of the test, and no operators reported of any related symptoms. At the end of the test, operators were rated on a 6-point scale according to the severity of symptoms (scale: 0 = none, 1 = minor symptoms, 2 = symptoms but not significant, 3 = significant symptoms, 4 = severe symptoms, and 5 = severe to overwhelming).

**Statistical Considerations**

Statistical analyses were performed by using SPSS 26.0 software (IBM, USA). The Shapiro‒Wilk test was used to determine the normality of the measurement data. Normally distributed variables are expressed as the mean ± standard deviation. Differences in the measured parameters before and after the operation were evaluated by using paired t tests, and the intergroup differences were compared by using independent sample t tests. Nonnormally distributed data are expressed as medians and interquartile ranges (M [Q1, Q3]), and the Wilcoxon signed-rank test was used for intragroup difference evaluation, whereas the Mann‒Whitney U test was used for intergroup difference evaluation. Count data are expressed as frequencies and percentages, and differences between two groups were compared by using the chi-square χ2 test. Fisher's exact test was used when the theoretical frequency was < 0.05. When the test level was α= 0.05, P < 0.05 was considered to indicate a statistically significant difference.
